# Discharge neutrophily-to-lymphocyte ratio and its trajectory as predictors of 30-day outcomes in acute heart failure

**DOI:** 10.1093/eschf/xvag059

**Published:** 2026-02-19

**Authors:** Gil Marcus, Shiri L Maymon, Eran Kalmanovich, Gil Moravsky, Ido Minha, Avishay Grupper, Anat Milman, Shmuel Fuchs, Sa’ar Minha

**Affiliations:** Department of Cardiology, Shamir Medical Center, Be'er Ya'akov 70300, Israel; Gray Faculty of Medical and Health Sciences, Tel-Aviv University, P.O. Box 39040, Tel-Aviv 6997801, Israel; Gray Faculty of Medical and Health Sciences, Tel-Aviv University, P.O. Box 39040, Tel-Aviv 6997801, Israel; Department of Otolaryngology, Tel-Aviv Sourasky Medical Center, Tel-Aviv 6423906, Israel; Department of Cardiology, Shamir Medical Center, Be'er Ya'akov 70300, Israel; Gray Faculty of Medical and Health Sciences, Tel-Aviv University, P.O. Box 39040, Tel-Aviv 6997801, Israel; Department of Cardiology, Shamir Medical Center, Be'er Ya'akov 70300, Israel; Gray Faculty of Medical and Health Sciences, Tel-Aviv University, P.O. Box 39040, Tel-Aviv 6997801, Israel; The Rachel and Selim Benin School of Computer Science and Engineering, The Hebrew University of Jerusalem, Jerusalem, Israel; Department of Cardiology, Shamir Medical Center, Be'er Ya'akov 70300, Israel; Gray Faculty of Medical and Health Sciences, Tel-Aviv University, P.O. Box 39040, Tel-Aviv 6997801, Israel; Department of Cardiology, Shamir Medical Center, Be'er Ya'akov 70300, Israel; Gray Faculty of Medical and Health Sciences, Tel-Aviv University, P.O. Box 39040, Tel-Aviv 6997801, Israel; Department of Cardiology, Shamir Medical Center, Be'er Ya'akov 70300, Israel; Gray Faculty of Medical and Health Sciences, Tel-Aviv University, P.O. Box 39040, Tel-Aviv 6997801, Israel; Department of Cardiology, Shamir Medical Center, Be'er Ya'akov 70300, Israel; Gray Faculty of Medical and Health Sciences, Tel-Aviv University, P.O. Box 39040, Tel-Aviv 6997801, Israel

**Keywords:** Neutrophil-to-lymphocyte ratio, Acute decompensated heart failure, Discharge biomarkers, 30-day readmission, 30-day mortality

## Abstract

**Aims:**

To evaluate whether discharge neutrophil-to-lymphocyte ratio (NLR) and its in-hospital trajectory predict 30-day outcomes after acute decompensated heart failure (ADHF) hospitalization, and to compare discharge NLR with admission NLR and with serial NLR measurement.

**Methods:**

Retrospective cohort of 6784 ADHF discharges (2007–17; median age 78 [interquartile range: 69–85] years; 48.8% women). Patients were classified by discharge NLR (<5 vs ≥5) and by NLR trajectory (low→low, low→high, high→low, high→high). Primary endpoints were 30-day all-cause readmission and 30-day all-cause mortality. Multivariable Cox models adjusted for age, sex, anaemia, chronic kidney disease, diabetes, ischaemic heart disease, atrial fibrillation, peripheral vascular disease, and COPD. Discrimination was assessed using areas under the curve (AUCs) from adjusted logistic models.

**Results:**

High discharge NLR (≥5) was present in 2258/6784 (33.3%). Event rates were higher with high vs low discharge NLR for readmission (25.5% vs 19.7%, *P* < .001) and mortality (7.8% vs 2.8%, *P* < .001). High discharge NLR was independently associated with readmission (hazard ratio [HR] 1.21, 95% confidence interval [CI] 1.05–1.40, *P* = .007) and mortality (HR 1.92, 95% CI 1.46–2.53, *P* < .001). Trajectory further stratified risk: high→high had the greatest risk (readmission HR 1.42, 95% CI 1.25–1.62, *P* < .001; mortality HR 3.42, 95% CI 2.53–4.62, *P* < .001); low→high was also high-risk (readmission HR 1.46, 95% CI 1.20–1.77, *P* < .001; mortality HR 3.05, 95% CI 2.00–4.65, *P* < .001). High→low showed reduced but residual risk vs low→low (readmission HR 1.07, 95% CI .93–1.23, *P* = .321; mortality HR 1.52, 95% CI 1.06–2.16, *P* = .021). Discharge NLR outperformed admission NLR (mortality AUC 0.731 vs 0.705; readmission AUC 0.573 vs 0.564). Serial NLR added minimal discrimination beyond discharge NLR alone (mortality AUC 0.736 vs 0.734; readmission AUC 0.571 for both).

**Conclusions:**

Discharge NLR is an independently prognostic, routinely available biomarker for 30-day readmission and mortality after ADHF. Persistently elevated or rising NLR identifies patients at the highest short-term risk, while normalization attenuates but does not eliminate risk. A single discharge measurement performs comparably to serial assessment, supporting practical integration of discharge NLR into risk-stratified follow-up, including in resource-limited settings.

## Introduction

Acute decompensated heart failure (ADHF) marks a high-risk phase in the clinical course of heart failure (HF), with substantial rates of early readmission and death following discharge.^1^ Identifying patients at greatest risk during this vulnerable transition remains a key challenge in HF care.^2^ To guide decisions regarding discharge timing, follow-up intensity, and post-discharge support, clinicians often rely on biomarkers such as B-type natriuretic peptide or N-terminal pro–B-type natriuretic peptide, measured serially during admission, to assess improvement and residual risk.^3^ In resource-constrained health systems, where such tests may be unavailable or unaffordable, the difficulty of optimizing discharge planning and targeting follow-up is therefore greater.^1^ This diagnostic gap likely contributes to the observed inequities in HF outcomes across healthcare systems with differing levels of resources.^4^

To improve diagnostic equity, the World Health Organization (WHO) developed the Model List of Essential In Vitro Diagnostics, which promotes the use of simple, scalable tests suitable for all levels of care.^5^ Among these, the complete blood count (CBC) is nearly universally available and routinely obtained during HF hospitalizations. From this standard panel, the neutrophil-to-lymphocyte ratio (NLR)—a marker that reflects the balance between innate and adaptive immune activity—can be easily calculated. First proposed as a prognostic tool in oncology nearly two decades ago,^6^ NLR has since been widely investigated across numerous clinical settings, including sepsis,^7^ critical illness,^8^ autoimmune conditions,^9^ and cardiovascular disease.^10^ In these contexts, elevated NLR has consistently been associated with poor clinical outcomes and is thought to reflect systemic inflammatory stress.

In HF specifically, elevated NLR at admission has been linked to worse prognosis in several cohorts. However, its utility during the course of hospitalization remains underexplored. In particular, the prognostic significance of discharge NLR and the changes in NLR levels between admission and discharge have not been systematically examined. Whether persistent elevation at discharge reflects unresolved inflammatory risk—or whether a declining trajectory signals adequate clinical recovery—remains uncertain. Clarifying these relationships could enhance discharge decision-making and optimize follow-up strategies, particularly in resource-limited settings.

To address this gap, we evaluated the prognostic value of NLR measured at both admission and discharge in a well-characterized cohort of patients hospitalized with ADHF. Specifically, we examined whether discharge NLR—and its trajectory during hospitalization—were associated with short-term outcomes, including 30-day readmission and all-cause mortality.

## Methods

### Study design and population

This retrospective cohort study analysed consecutive adults (≥18 years) hospitalized with ADHF at Shamir Medical Center, Israel, from 1 January 2007 through 31 December 2017, identified by primary discharge ICD-9 codes 428.xx, 429.xx, or 514. Patients were eligible if they had a CBC with differential at admission and discharge, allowing calculation of the NLR and its trajectory from admission to discharge. Patients with a lymphocyte count of zero were excluded because an NLR could not be computed with a value of zero in the denominator. Patients who died during the index hospitalization were also excluded to comply with the focus of the analysis on post-discharge outcomes. Demographic, clinical, and laboratory data were extracted from the hospital’s electronic medical record, and vital-status information was cross-checked against the Israeli Ministry of Interior registry. The study was approved by the Shamir Medical Center Institutional Review Board with a waiver of individual informed consent owing to its retrospective design.

### NLR grouping, cut-off definition, and study outcomes

The association between NLR and outcomes was examined using two complementary approaches. For descriptive statistics, univariate comparisons, and transition from admission to discharge (low → low, low → high, high → low, high → high) analysis, NLR was dichotomized into ‘high’ and ‘low’ categories by which patients were grouped.

A universally accepted cut-off for ‘high’ NLR has not yet been established. Published studies report thresholds clustering between four and six, derived from tertile- or quartile-split boundaries,^11,12^ maximally selected statistics,^13^ or receiver operating characteristics (ROC) curve–based Youden indices.^14^ A recent meta-analysis of 18 231 patients suggested a mean study-level cut-off of 4.7,^15^ whereas some cohorts have favoured the integer 5.0 for its mnemonic simplicity.^11,16,17^ To determine the most suitable threshold for our cohort, we first constructed an ROC curve for 30-day mortality; the Youden index identified an optimum of 4.93 (Supplementary Figure S1). Comparing this data-driven value with the literature-based 4.7 and the integer 5.0 revealed virtually identical performance (sensitivity 0.60–0.63, specificity 0.64–0.67, accuracy 0.66–0.67, univariate OR ≈ 3.0; Supplementary Table S1). Given its ease of clinical recall and comparable discrimination, we adopted NLR ≥ 5 as the working definition of a high NLR.

In parallel, to preserve the full informational value of the variable, discharge NLR was also modelled as a continuous covariate in all multivariable analyses.

The study’s primary outcomes were 30-day all-cause readmission and 30-day all-cause mortality, assessed from the date of hospital discharge.

### Statistical analysis

Continuous variables were evaluated for normality using the Shapiro–Wilk test. Variables with normal distribution are presented as mean ± standard deviation and were compared using the independent samples *t*-test, while non-normally distributed variables are expressed as medians with interquartile ranges and were compared using the Mann–Whitney *U* test. Categorical variables are reported as counts and percentages and were compared using the chi-squared test. Kaplan–Meier survival curves with log-rank testing were used to evaluate unadjusted associations between discharge NLR groups and 30-day outcomes.

Multivariable Cox proportional hazards regression was used to examine the independent association between NLR and 30-day readmission and mortality. Hazard ratios (HR) and 95% confidence intervals (CIs) were derived for both discharge NLR and admission-to-discharge NLR trajectory. Covariates were selected a priori and forced into all models based on their known association with outcomes^18^ in acute HF. These included age,^19^ sex,^20^ anaemia,^21^ chronic kidney disease (CKD),^22^ diabetes mellitus (DM),^23^ ischaemic heart disease (IHD),^24^ atrial fibrillation (Afib),^25^ peripheral vascular disease (PVD),^26^ and chronic obstructive pulmonary disease (COPD).^27^ Adjusted survival curves for NLR trajectory groups were derived from the multivariable model.

Additional exploratory analyses were performed to evaluate the prognostic utility of serial NLR compared with single-point measurements. All analyses used multivariable logistic regression models adjusted for the same covariates described above. First, in the sub-cohort of patients with high NLR at admission, we assessed whether a reduction to low NLR at discharge was associated with improved outcomes. Second, to evaluate the discriminative value of serial NLR compared with discharge NLR alone, ROC curves and area under the curve (AUC) values were generated from the adjusted models. Lastly, we compared models including only discharge or only admission NLR to determine which single time point better predicted 30-day outcomes.

All analyses were performed using R software version 4.2.0 (R Foundation for Statistical Computing, Vienna, Austria) and Python version 3.10. A two-tailed *P*-value <.05 was considered statistically significant.

## Results

**Figure 1:**
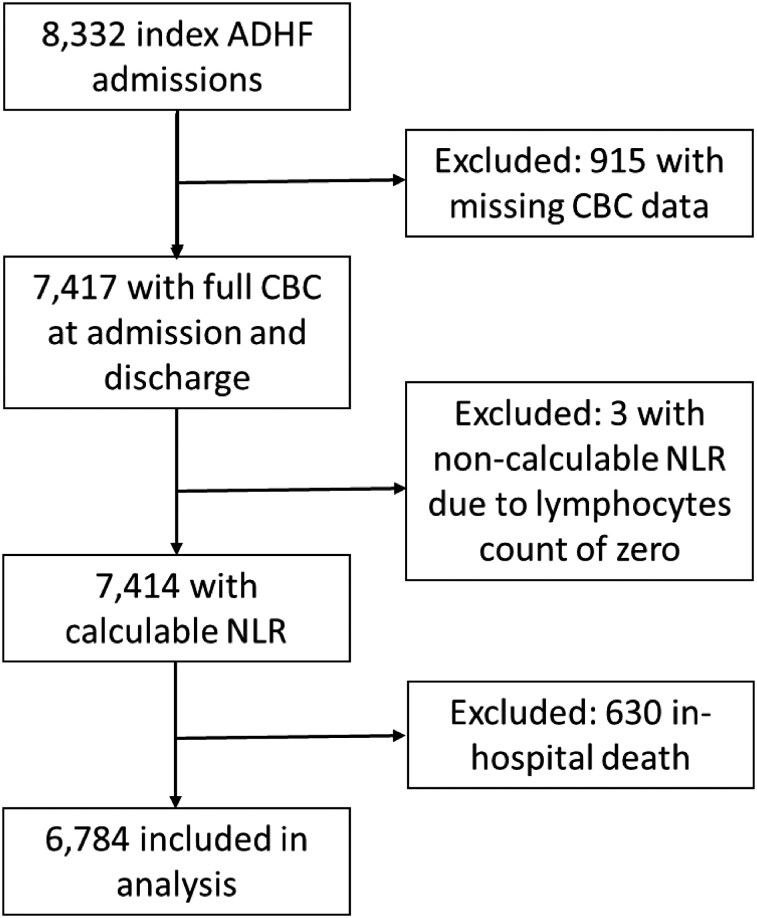
Flow diagram of patient selection. Flow of patient inclusion for the analytic cohort. From 8332 index hospitalizations for ADHF, patients were excluded for missing CBC data, zero lymphocyte counts, or in-hospital death, yielding a final sample of 6784 with valid pre- and post-hospitalization NLR. ADHF, acute decompensated heart failure; CBC, complete blood count; NLR, neutrophil-to-lymphocyte ratio

Of 8332 index admissions for ADHF during the study period, 7417 (89.0%) patients had a CBC with differential at admission and discharge. Three patients (0.04%) were excluded due to a lymphocyte count of zero, and 630 (8.5%) were excluded due to in-hospital death, yielding a final analytic cohort of 6784 patients (*Fig. 1*). Among the 6784 patients included in the final analytic cohort, 2258 (33.3%) were classified as having a high discharge NLR (≥5). Compared with patients in the low NLR group, those with high discharge NLR were older (median 79 vs 77 years, *P* < .001) and less likely to be female (46.6% vs 51.4%, *P* < .001). Several comorbidities were more common in the high NLR group: anaemia (70.7% vs 64.1%, *P* < .001), CKD (36.1% vs 30.1%, *P* < .001), and COPD (19.4% vs 13.9%, *P* < .001), while rates of DM, IHD, AFib, and PVD were similar between groups (*Table 1*). Laboratory parameters at discharge reflected more advanced disease among patients with high NLR, including elevated leukocyte counts (10.0 vs 8.7 K/µl, *P* < .001), urea (56.5 vs 46.0 mg/dl, *P* < .001), and creatinine (1.2 vs 1.1 mg/dl, *P* < .001), and lower haemoglobin (11.6 ± 2.1 vs 11.9 ± 2.0 g/dl, *P* < .001). Left ventricular ejection fraction distribution was similar between groups (*P* = .97), with no significant differences observed across EF categories.

**Figure 2:**
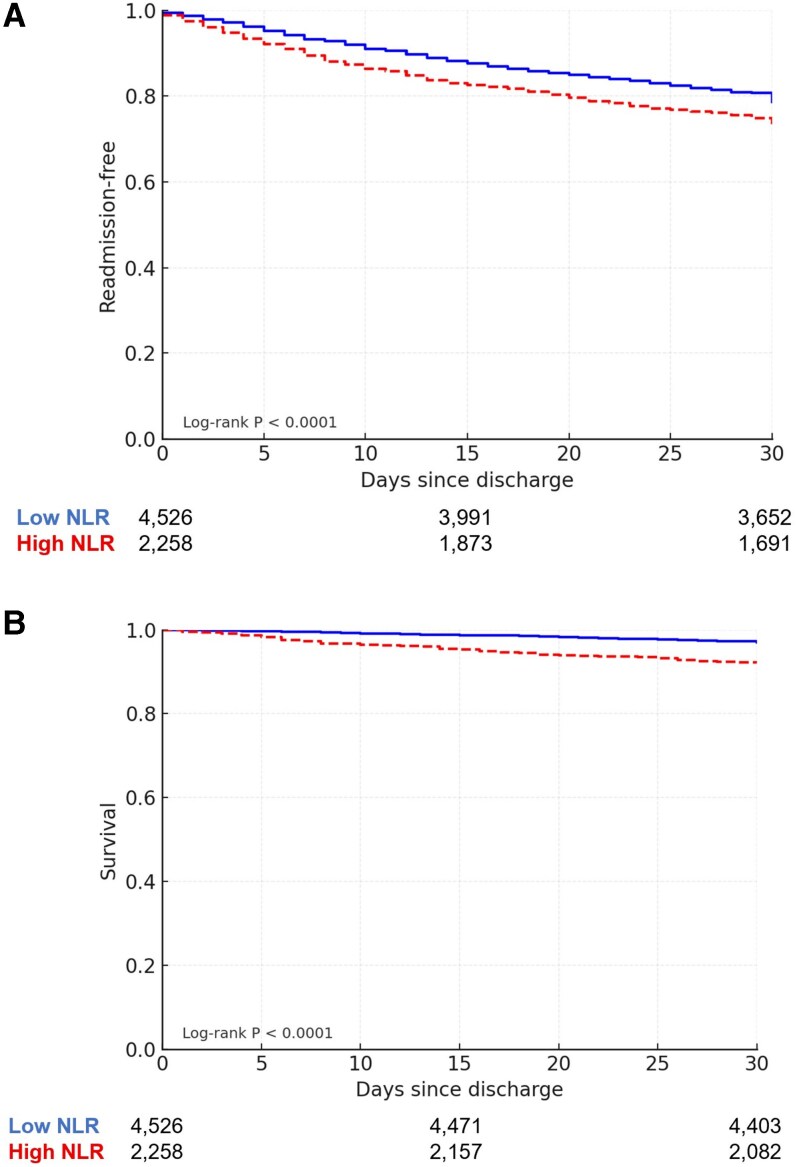
Kaplan–Meier curve for 30-day outcomes by discharge NLR. (A) A 30-day readmission by discharge NLR; (B) 30-day mortality by discharge NLR. Cumulative incidence of 30-day readmission (panel A) and 30-day mortality (panel B) following discharge, stratified by NLR group (<5 vs ≥5). Log-rank *P*-values shown. NLR, neutrophil-to-lymphocyte ratio

**Table 1:** Baseline characteristics by discharge NLR group

Variable	Low NLR (*N* = 4526)	High NLR (*N* = 2258)	*P* value
Age, years, median [IQR]	77.0 [67.0–84.0]	79.0 [71.0–86.0]	<.001
Female sex, *n* (%)	2327 (51.4)	1052 (46.6)	<.001
Medical history, *n* (%)			
Hypertension	825 (18.2)	393 (17.4)	.424
Diabetes mellitus	2325 (51.4)	1113 (49.3)	.112
Obesity	1 025 (22.6)	433 (19.2)	.001
Smoking	792 (17.5)	358 (15.9)	.096
Ischaemic heart disease	1816 (40.1)	874 (38.7)	.272
Atrial fibrillation	1393 (30.8)	721 (31.9)	.348
COPD	627 (13.9)	438 (19.4)	<.001
Peripheral vascular disease	296 (6.5)	165 (7.3)	.258
Chronic kidney disease	1364 (30.1)	815 (36.1)	<.001
Anaemia	2869 (64.1)	1575 (70.7)	<.001
Chronic medications, *n* (%)			
Alpha-blockers	464 (10.3)	253 (11.2)	.246
Beta-blockers	1633 (36.1)	836 (37.0)	.463
Calcium-channel blockers	1090 (24.1)	609 (27.0)	.011
ACE inhibitor	857 (18.9)	384 (17.0)	.057
Angiotensin-receptor blocker	528 (11.7)	236 (10.5)	.147
Aldosterone antagonist	85 (1.9)	69 (3.1)	.003
Antiarrhythmics	272 (6.0)	170 (7.5)	.019
Anti-platelets	1914 (42.3)	897 (39.7)	.046
Oral anticoagulants	623 (13.8)	314 (13.9)	.903
Statins	1857 (41.0)	829 (36.7)	<.001
Digoxin	219 (4.8)	104 (4.6)	.716
Loop diuretics	2389 (52.8)	1240 (54.9)	.102
Laboratory indices, median [IQR]			
Haemoglobin, mean ± SD (g/dL)	11.9 ± 2.0	11.6 ± 2.1	<.001
WBC, K/µL	8.7 [6.8–11.4]	10.0 [7.9–13.1]	<.001
Urea, mg/dL	46.0 [34.7–66.7]	56.5 [40.0–85.3]	<.001
Sodium, mmol/L	138.0 [135.0–141.0]	138.0 [134.0–140.6]	<.001
Creatinine, mg/dL	1.1 [0.8–1.4]	1.2 [0.9–1.7]	<.001
Left-ventricular ejection fraction by echocardiography, *n* (%)			.97
Preserved (EF ≥ 50%)	815 (18.0)	342 (15.1)	
Mildly reduced (EF 40–49%)	266 (5.9)	105 (4.7)	
Moderately reduced (EF 30–39%)	441 (9.7)	182 (8.1)	
Severely reduced (EF < 30%)	199 (4.4)	84 (3.7)	

CKD, chronic kidney disease; COPD, chronic obstructive pulmonary disease; EF, ejection fraction; IQR, interquartile range; NLR, neutrophil-to-lymphocyte ratio; SD, standard deviation; PVD, peripheral vascular disease.

During the index hospitalization, patients with high NLR were less likely to undergo percutaneous coronary intervention (7.7% vs 10.5%, *P* < .001) or diagnostic angiography (4.5% vs 5.8%, *P* = .036), and more likely to require dialysis (1.8% vs 0.8%, *P* < .001) (*Table 2*). At discharge, guideline-recommended therapies such as angiotensin-converting enzyme (ACE) inhibitors (27.1% vs 32.4%, *P* < .001), statins (56.1% vs 63.1%, *P* < .001), and beta-blockers (59.5% vs 62.0%, *P* = .050) were prescribed less frequently in the high-NLR group. Median length of HF hospitalization was longer among patients with high NLR at discharge (6 vs 5 days, *P* < .001).

**Table 2:** In-hospital interventions and discharge medications by NLR group

Variable	Low NLR (*N* = 4526)	High NLR (*N* = 2258)	*P*-value
Interventions during index admission, *n* (%)			
Diagnostic coronary angiography	261 (5.8)	102 (4.5)	.036
Percutaneous coronary intervention	475 (10.5)	174 (7.7)	<.001
Pacemaker implantation	68 (1.5)	19 (0.8)	.03
Cardiac resynchronization therapy	10 (0.2)	4 (0.2)	.928
Coronary artery bypass grafting	109 (2.4)	55 (2.4)	1
Dialysis	38 (0.8)	41 (1.8)	<.001
Medications at discharge, *n* (%)			
Alpha-blockers	691 (15.3)	377 (16.7)	.137
Beta-blockers	2805 (62.0)	1343 (59.5)	.05
Calcium-channel blockers	1503 (33.2)	850 (37.6)	<.001
ACE inhibitors	1465 (32.4)	611 (27.1)	<.001
Angiotensin receptor blocker	748 (16.5)	321 (14.2)	.015
Aldosterone antagonist	113 (2.5)	82 (3.6)	.01
Antiarrhythmics	446 (9.9)	244 (10.8)	.238
Anti-platelets	2945 (65.1)	1353 (59.9)	<.001
Oral anticoagulants	1315 (29.1)	630 (27.9)	.336
Statins	2854 (63.1)	1266 (56.1)	<.001
Digoxin	219 (4.8)	115 (5.1)	.716
Loop diuretics	3637 (80.4)	1832 (81.1)	.466

ACE, angiotensin-converting enzyme; ARB, angiotensin receptor blocker; CABG, coronary artery bypass graft; CRT, cardiac resynchronization therapy; NLR, neutrophil-to-lymphocyte ratio; PCI, percutaneous coronary intervention.

### Discharge NLR and risk of 30-day outcomes

Both primary outcomes were significantly more frequent among patients with high discharge NLR: 30-day readmission occurred in 25.5% vs 19.7% (*P* < .001), and 30-day mortality in 7.8% vs 2.8% (*P* < .001) (*Figure 2*). The combined rate of either readmission or death within 30 days was also higher in the high-NLR group (29.3% vs 20.9%, *P* < .001) (*Table 3*).

**Table 3:** Unadjusted outcomes by discharge NLR group

Outcome, *n* (%)	Low NLR (*N* = 4 526)	High NLR (*N* = 2 258)	*P* value
Length of stay, days, median [IQR]	5 [3–9]	6 [3–10]	<.001
Readmission within 30 days	892 (19.7)	575 (25.5)	<.001
Mortality within 30 days	126 (2.8)	177 (7.8)	<.001
Readmission or mortality within 30 days	948 (20.9)	662 (29.3)	<.001

NLR, neutrophil-to-lymphocyte ratio; IQR, interquartile range.

In multivariable analysis, high discharge NLR remained independently associated with both 30-day readmission (HR 1.21, 95% CI 1.05–1.40, *P* = .007) and 30-day mortality (HR 1.92, 95% CI 1.46–2.53, *P* < .001), even after adjustment for age, sex, and clinical covariates (*Table 4*). Similar associations were observed when NLR was modelled as a continuous variable.

**Table 4:** Adjusted hazard ratios for 30-day outcomes by discharge NLR

Predictor	30-day readmission hR (95% cI, *P*)	30-day mortality hR (95% CI, *P*)
High (≥5) discharge NLR	1.21 (1.05–1.40, *P* = .007)	1.92 (1.46–2.53, *P* < .001)
Discharge NLR, continuous^a^	1.02 (1.01–1.04, *P* = .002)	1.06 (1.04–1.08, *P* < .001)
Age^b^	1.00 (1.00–1.01, *P* = .442)	1.05 (1.04–1.07, *P* < .001)
Anaemia	1.21 (1.07–1.36, *P* = .002)	1.31 (1.00–1.71, *P* = .048)
COPD	1.03 (0.90–1.18, *P* = .618)	0.62 (0.43–0.89, *P* = .010)
Chronic kidney disease	0.99 (0.89–1.11, *P* = .907)	0.66 (0.50–0.87, *P* = .003)
Atrial fibrillation	0.99 (0.88–1.10, *P* = .823)	0.69 (0.54–0.90, *P* = .005)
Ischaemic heart disease	1.01 (0.90–1.12, *P* = 0.925)	0.80 (0.62–1.02, *P* = .075)
Diabetes mellitus	0.96 (0.86–1.07, *P* = .458)	0.90 (0.70–1.14, *P* = .375)
Peripheral vascular disease	1.00 (0.82–1.22, *P* = .963)	0.91 (0.54–1.55, *P* = .739)
Female sex	1.00 (0.90–1.12, *P* = .960)	1.25 (0.98–1.59, *P* = .074)

^a^Per increase in 1 unit of NLR.

^b^Per increase in 1 year of age.

CI, confidence interval; CKD, chronic kidney disease; COPD, chronic obstructive pulmonary disease; HR, hazard ratio; NLR, neutrophil-to-lymphocyte ratio; PVD, peripheral vascular disease.

**Figure 3:**
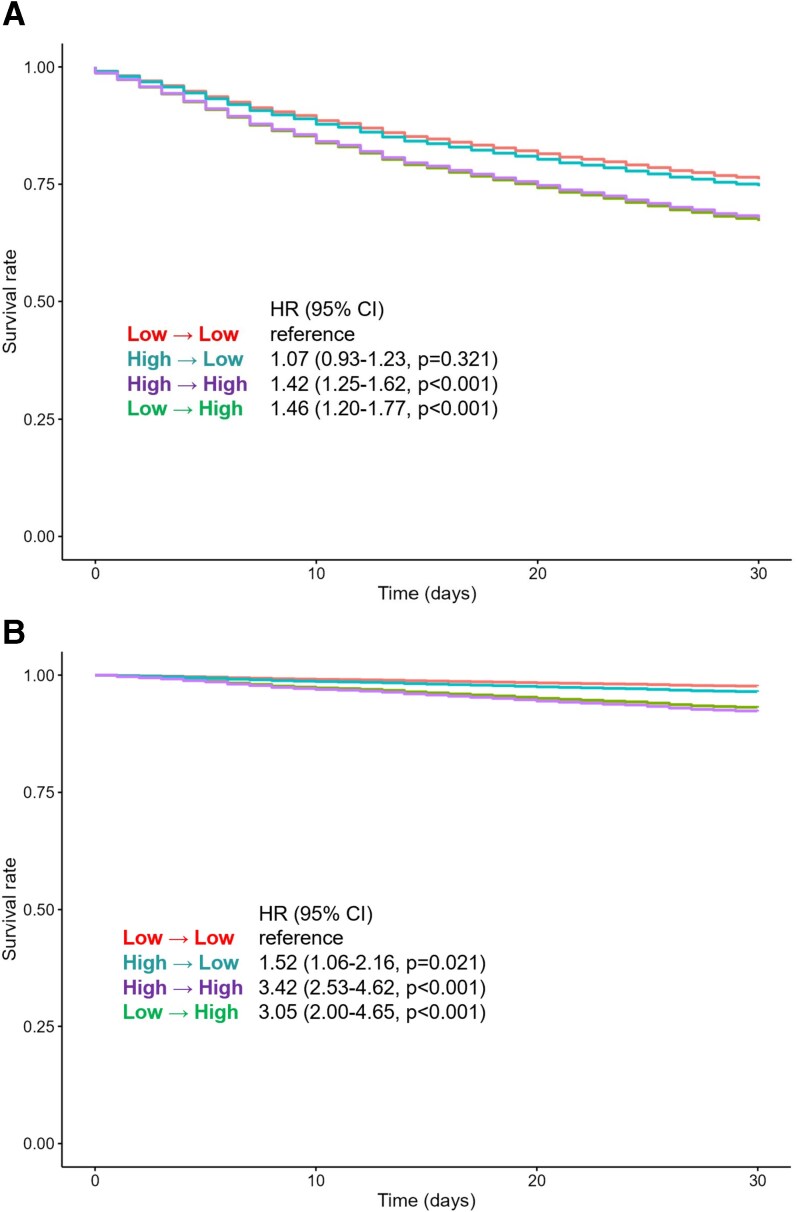
Adjusted (derived from Cox model) 30-day outcomes by NLR dynamic: (A) A 30-day readmission by NLR dynamic; (B) 30-day mortality by NLR dynamic. Adjusted survival estimates for 30-day readmission based on NLR trajectory from admission to discharge (low→low, low→high, high→low, high→high), derived from Cox regression. CI, confidence interval; HR, hazard ratio; NLR, neutrophil-to-lymphocyte ratio;

Dynamic changes in NLR from admission to discharge further stratified risk (*Figure 3*). Patients who remained in the low NLR group throughout hospitalization (low→low) had the most favourable outcomes and served as the reference group in trajectory analyses. Notably, patients whose NLR rose from low at admission to high at discharge (low→high) had markedly elevated risk of 30-day readmission (HR 1.46, 95% CI 1.20–1.77, *P* < .001) and mortality (HR 3.05, 95% CI 2.00–4.65, *P* < .001), approaching the risk observed in the persistently high group (high→high: readmission HR 1.42, 95% CI 1.25–1.62, *P* < .001; mortality HR 3.42, 95% CI 2.53–4.62, *P* < .001). In contrast, patients whose NLR declined from high to low (high→low) showed a meaningful reduction in risk, though not to the level of the reference group (readmission HR 1.07, 95% CI 0.93–1.23, *P* = .321; mortality HR 1.52, 95% CI 1.06–2.16, *P* = .021).

### Serial versus single discharge NLR: exploratory analyses

Receiver operating characteristic curves derived from adjusted logistic regression models showed no added discriminative value for serial NLR over a single discharge NLR. For 30-day readmission, AUC remained 0.571 for both models; for 30-day mortality, AUC increased only marginally from 0.734 to 0.736 (Supplementary Figure S2).

When discharge and admission NLR were compared as single predictors in adjusted models, discharge NLR showed superior performance. For 30-day readmission, discharge NLR yielded an adjusted odds ratio (OR)s of 1.35 (95% CI 1.19–1.53, *P* < .001) with model AUC 0.573, compared with OR 1.16 (95% CI 1.03–1.31, *P* = .012) and AUC 0.564 for admission NLR. For 30-day mortality, discharge NLR showed an adjusted OR of 2.85 (95% CI 2.24–3.62, *P* < .001) and AUC 0.731, vs 1.93 (95% CI 1.50–2.48, *P* < .001) and AUC 0.705 for admission NLR (Supplementary Table S2).

## Discussion

In this large retrospective cohort study of patients hospitalized for ADHF, we found that discharge NLR was independently associated with both 30-day readmission and mortality, that it outperformed admission NLR in prognostic performance, and that improvement in NLR during hospitalization was associated with lower risk compared to persistently high values or increase NLR levels during hospitalization, though still higher than in patients with consistently low NLR.

Most prior studies demonstrating that elevated NLR predicts adverse outcomes in HF have focused on values measured at admission. One study by Shi et al., conducted in a cohort of 368 patients with HF with reduced ejection fraction (HFrEF), found that discharge NLR, but not admission NLR, was independently associated with long-term mortality; however, short-term outcomes such as 30-day readmission or death were not reported.^28^ Similarly, Boralkar et al. analysed admission NLR and its trajectory to discharge in 443 patients with HF with preserved ejection fraction (HFpEF) and found both to be predictive of long-term mortality, with trajectory showing stronger association; but again, no short-term outcomes were reported.^29^ To our knowledge, our study is the first to evaluate discharge NLR as a predictor of 30-day outcomes, and its large sample size, including patients with HF and reduced or preserved EF, provided sufficient power to assess short-term risks with greater precision. This focus on 30-day readmission and mortality is justified by three considerations: first, early post-discharge events following ADHF hospitalization have been recognized as a major public health priority;^30,31^ second, from a pathophysiological perspective, disturbances in the innate-to-adaptive inflammatory balance are more plausibly linked to imminent rather than distant clinical deterioration (although lower NLR values may also reflect a chronic inflammatory state, which may explain the association with long-term outcomes and chronic diseases);^32^ and third, prior studies linking admission NLR to outcomes have reported on early endpoints,^33^ further supporting a parallel approach when evaluating NLR at the time of discharge.

Changes in NLR from admission to discharge provided additional prognostic stratification. Patients whose elevated NLR at admission declined to normal levels by discharge had better outcomes than those with persistently high NLR, suggesting that a downward inflammatory trajectory may reflect meaningful clinical recovery and help refine discharge risk assessment. However, their risk remained higher than that of patients with consistently low NLR throughout hospitalization, indicating that early inflammation may leave a residual prognostic imprint. This is consistent with prior studies demonstrating that admission NLR alone is associated with worse outcomes in HF, regardless of its subsequent course.^33^ One exception is the aforementioned study by Shi et al., which found an association between discharge NLR and long-term mortality in HFrEF patients, which found an association between discharge NLR and long-term mortality in HFrEF patients, but not between admission NLR and outcomes.^28^ This discrepancy may reflect differences in statistical power, as their cohort included only 368 patients, compared with 6784 in our study, and thus may have been underpowered to detect the weaker association of admission NLR, while still sufficient to identify the stronger signal associated with discharge NLR.

While these results support the clinical relevance of NLR trajectory, our analyses suggest that its added prognostic value beyond discharge NLR alone may be limited. When assessed in terms of model discrimination, incorporating both admission and discharge NLR yielded only marginal improvement over discharge values alone—for example, the AUC for 30-day mortality increased from 0.734 to 0.736. These findings indicate that while serial NLR conveys biologically meaningful information, a single discharge measurement may be sufficient for prediction. Nonetheless, since CBCs are routinely performed at both admission and discharge for a variety of clinical reasons, the use of serial NLR incurs no additional cost or effort, making its inclusion in clinical risk assessments both justified and practical.

Notably, discharge NLR demonstrated stronger prognostic value for 30-day mortality than for readmission, among the two outcomes evaluated. The adjusted hazard ratio for mortality was over 50% greater than that for readmission, and model discrimination was substantially better for mortality. This discrepancy likely reflects both biological and methodological factors. Mortality is more tightly coupled to physiological deterioration, which NLR—an integrative marker of systemic inflammatory stress—may capture more directly.^32^ In contrast, hospital readmission is a more heterogeneous endpoint, often driven by non-biological factors such as healthcare access, follow-up adherence, and social determinants of health,^4^ which dilute the predictive strength of clinical biomarkers. Moreover, the presence of a competing risk relationship may contribute to this pattern: patients who died within 30 days were, by definition, not at risk of readmission, and those at highest risk of mortality may have otherwise been readmitted had they survived.^34^ This dynamic underscores the importance of considering mortality and readmission not as independent but as interrelated outcomes.

Patients with elevated discharge NLR represented a clinically and biologically distinct subgroup—typically older, more often male, and burdened with comorbidities such as CKD, anaemia, and COPD, all of which are known drivers of systemic inflammation in hospitalized patients.^35^ Laboratory findings further reflected widespread physiological dysfunction and inflammatory stress. Notably, these patients were modestly less likely to receive guideline-directed medical therapy (GDMT) at discharge, including ACE inhibitors and beta-blockers—a pattern consistent with prior observations in which high NLR combined with under-treatment conferred worse outcomes.^36^ While this may in part reflect appropriate caution in patients with frailty or renal impairment, it also raises concern for therapeutic inertia—an increasingly recognized barrier to optimal care.^37^ The co-occurrence of elevated NLR and reduced GDMT use suggests that discharge NLR could serve not only as a prognostic marker, but also as a practical trigger for more deliberate discharge planning, medication optimization, and closer follow-up.

This study has several limitations. First, its retrospective, single-centre design may limit generalizability despite the large and diverse cohort. Second, important clinical variables were unavailable, including natriuretic peptide levels, objective markers of congestion, and social determinants of health—all of which may confound or mediate observed associations. Third, although NLR is biologically plausible and readily accessible, it remains a non-specific marker of inflammation and may be affected by concurrent conditions such as infection, malignancy, or autoimmune disease. Finally, we lacked data on post-discharge factors such as medication adherence, outpatient follow-up, or rehospitalizations at other institutions, which may have influenced 30-day outcomes.

### Conclusion

In conclusion, discharge NLR is a simple, widely accessible, and independently prognostic marker of 30-day outcomes following hospitalization for ADHF, and it demonstrated superior predictive value compared to admission NLR. Patients with persistently elevated or incompletely normalized NLR constitute a high-risk subgroup that may benefit from targeted post-discharge strategies. These findings highlight the potential of routine laboratory data to inform transitional care and promote more equitable HF management across resource-diverse healthcare settings.

## Supplementary Material

xvag059_Supplementary_Data

## Declarations

### Data Availability

The data underlying this article cannot be shared publicly because they contain sensitive patient information from the electronic medical records of Shamir Medical Center. Data will be shared upon reasonable request to the corresponding author.
