# Supplementary material for: Discharge neutrophily-to-lymphocyte ratio and its trajectory as predictors of 30-day outcomes in acute heart failure
Source: ESC Heart Fail. 2026 Feb 19;13(2):xvag059. doi: 10.1093/eschf/xvag059 (PMC13122620; doi:10.1093/eschf/xvag059)
Supplement: xvag059_Supplementary_Data [file xvag059_supplementary_data.docx]

Discharge Neutrophil-to-Lymphocyte Ratio and Its Trajectory as Predictors of 30-Day Outcomes in Acute Heart Failure
supplementary material

Supplementary Table S1. Operating Characteristics of Discharge Neutrophil-to-Lymphocyte Ratio Cut-Offs For 30-Day Mortality

| Metric | 4.7 | 4.93 | 5 | Δ (max – min) |
| --- | --- | --- | --- | --- |
| Sensitivity | 0.63 | 0.6 | 0.6 | 0.03 |
| Specificity | 0.64 | 0.67 | 0.67 | 0.03 |
| Positive Predictive Value | 0.08 | 0.08 | 0.08 | < 0.01 |
| Negative Predictive Value | 0.97 | 0.97 | 0.97 | < 0.01 |
| Accuracy | 0.66 | 0.67 | 0.67 | 0.01 |
| Odds Ratio (95 % CI) | 2.93 (2.31 – 3.72) | 3.00 (2.38 – 3.80) | 2.97 (2.35 – 3.76) |  |

CI = Confidence interval.

Supplementary Table S2. Adjusted Odds Ratios and Model Discrimination for Admission vs. Discharge NLR

|  | 30-Day Readmission | | AUC | 30-Day Mortality | | AUC |
| --- | --- | --- | --- | --- | --- | --- |
|  | Adjusted OR (95% CI) | *p*-value |  | Adjusted OR (95% CI) | *p*-value |  |
| Admission NLR | 1.16 (1.03–1.31) | 0.012 | 0.564 | 1.93 (1.50–2.48) | <0.001 | 0.705 |
| Discharge NLR | 1.35 (1.19–1.53) | <0.001 | 0.573 | 2.85 (2.24–3.62) | <0.001 | 0.731 |

NLR = neutrophil-to-lymphocyte ratio; OR = odds ratio; CI = confidence interval; AUC = area under the curve.

Supplementary Figure S1. Discharge Neutrophil-to-Lymphocyte Ratio: Receiver Operating Characteristic curves for 30-Day Mortality vs 30-Day Readmission


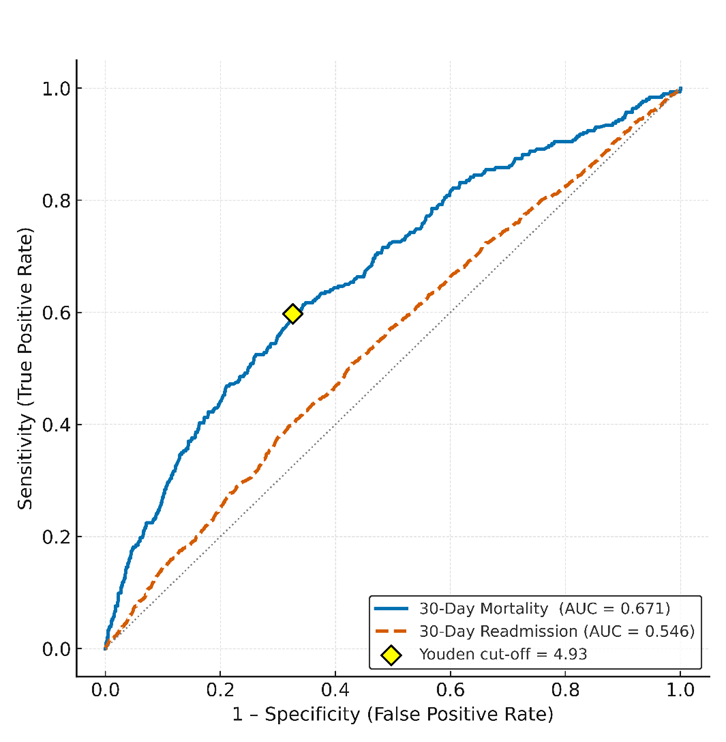


The solid blue curve shows moderate discrimination of NLR for 30-day all-cause mortality (AUC 0.671, 95 % CI 0.639–0.702). The dashed orange curve demonstrates only limited discrimination for 30-day readmission (AUC 0.546, 95 % CI 0.519–0.574). The yellow diamond marks the Youden-optimized threshold (NLR = 4.93), used as the basis for the ≥ 5 “high-NLR” category in subsequent analyses (sensitivity 0.60, specificity 0.67). NLR at discharge offers clinically relevant accuracy for short-term mortality but adds little value for predicting early readmission, supporting its use primarily as a prognostic rather than rehospitalization marker.

Supplementary Figure S2. Prognostic Performance of Serial Versus Single-Point NLR Measurement for 30-Day Outcomes


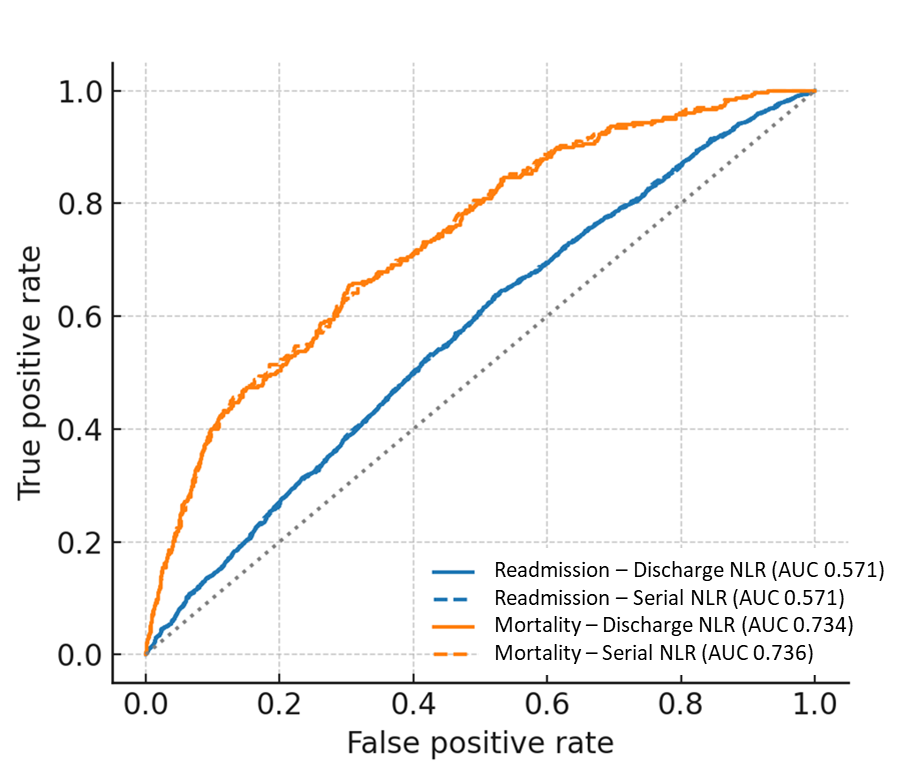


Legend: Receiver Operating Characteristic curves derived from logistic regression models adjusted for age, sex, and clinical covariates, comparing serial NLR (admission and discharge) versus discharge NLR alone. Serial measurement of NLR shows minimal incremental discriminative value over a single discharge measurement for predicting 30-day mortality.
